# Compositional differences in simulated root exudates elicit a limited functional and compositional response in soil microbial communities

**DOI:** 10.3389/fmicb.2015.00817

**Published:** 2015-08-11

**Authors:** Michael S. Strickland, Rebecca L. McCulley, Jim A. Nelson, Mark A. Bradford

**Affiliations:** ^1^Department of Biological Sciences, Virginia Polytechnic Institute and State UniversityBlacksburg, VA, USA; ^2^Department of Plant and Soil Science, University of KentuckyLexington, KY, USA; ^3^School of Forestry and Environmental Studies, Yale UniversityNew Haven, CT, USA

**Keywords:** land cover, land use legacies, low molecular weight carbon compounds, microbial community function, microbial community composition, root exudates

## Abstract

Inputs of low molecular weight carbon (LMW-C) to soil – primarily via root exudates– are expected to be a major driver of microbial activity and source of stable soil organic carbon. It is expected that variation in the type and composition of LMW-C entering soil will influence microbial community composition and function. If this is the case then short-term changes in LMW-C inputs may alter processes regulated by these communities. To determine if change in the composition of LMW-C inputs influences microbial community function and composition, we conducted a 90 day microcosm experiment whereby soils sourced from three different land covers (meadows, deciduous forests, and white pine stands) were amended, at low concentrations, with one of eight simulated root exudate treatments. Treatments included no addition of LMW-C, and the full factorial combination of glucose, glycine, and oxalic acid. After 90 days, we conducted a functional response assay and determined microbial composition via phospholipid fatty acid analysis. Whereas we noted a statistically significant effect of exudate treatments, this only accounted for ∼3% of the variation observed in function. In comparison, land cover and site explained ∼46 and ∼41% of the variation, respectively. This suggests that exudate composition has little influence on function compared to site/land cover specific factors. Supporting the finding that exudate effects were minor, we found that an absence of LMW-C elicited the greatest difference in function compared to those treatments receiving any LMW-C. Additionally, exudate treatments did not alter microbial community composition and observable differences were instead due to land cover. These results confirm the strong effects of land cover/site legacies on soil microbial communities. In contrast, short-term changes in exudate composition, at meaningful concentrations, may have little impact on microbial function and composition.

## Introduction

One of the major ways that plants interact with soil microbial communities is via the exudation of low molecular weight carbon (LMW-C) compounds, composed primarily of sugars, amino acids, and organic acids (Grayston et al., 1997; Yang and Janssen, 2002; van Hees et al., 2005; Boddy et al., 2007; Phillips et al., 2011). LMW-C compounds are major constituents of root exudates (i.e., compounds that are actively or passively exuded from the root), as well as leachates of plant litter and other organic substances present in the environment (van Hees et al., 2005; de Graaff et al., 2010). These compounds are involved in the nutrient acquisition and/or stress reduction strategies of plants (Jones, 1998; Neumann and Roemheld, 2012). For example, the exudation of sugars may increase microbial activity in soil leading to increased plant available nitrogen (N; Drake et al., 2011; Phillips et al., 2011), organic acid exudation may solubilise phosphorus or chelate heavy metals (Strom et al., 2002; Bais et al., 2006; Nguyen, 2009; Marschner et al., 2011; Keiluweit et al., 2015), and exudation may counteract the mineral protection of soil C (Keiluweit et al., 2015). While all of these compounds play a role from the plant perspective, they also influence ecosystem processes, the surrounding soil, and soil communities (Bertin et al., 2003; Bronick and Lal, 2005; Dennis et al., 2010; Rukshana et al., 2011; Bais, 2012). In particular, root exudates have been shown to alter the composition of soil microbial communities and may alter the function of these communities as well (Hanson et al., 2008; Chapin et al., 2009; Eilers et al., 2010; Shi et al., 2011).

Given that LMW-C compounds can fuel upward of 50% of heterotrophic soil respiration, it is clear that soil microbial communities derive a significant amount of their C (and energy) from root exudates (van Hees et al., 2005). Indirect evidence of the importance of root exudates to these communities can be seen in studies that track the fate of recent photosynthate into the microbial biomass (Hogberg et al., 2008, 2010; Wu et al., 2010). For instance, Hogberg et al. (2008) showed that within 48 h recently fixed C had been taken up by microbes and remained within the microbial biomass for ∼15 days. Similarly, others have noted results consistent with this finding, suggesting that as much as 30–50% of belowground activity is fuelled by recent photosynthate (Hogberg et al., 2010; Wu et al., 2010; Bradford et al., 2012). Although the importance of root exudates as a source of C to the microbial community can be inferred from such findings, the ultimate impact of specific LMW-C compounds and combinations of these compounds on microbial community function cannot–in part because plants can alter the composition of exudates in response to changing environmental conditions (Dakora and Phillips, 2002; Marschner et al., 2011). Meaning that, the response of the microbial community to root exudates may be mediated by the specific identity, composition, and concentrations of LMW-C compounds being exuded by the plant at any point in time.

Evidence suggests that specific LMW compounds lead to changes in the composition of soil microbial communities. For instance, Eilers et al. (2010) showed that additions of LMW-C compounds, particularly an organic acid, led to shifts in microbial community composition, primarily due to increased relative abundance of those groups inferred to be *r*-strategists (i.e., Actinobacteria and Proteobacteria). Shi et al. (2011) also noted compositional shifts in the active component of the microbial community due to additions of various combinations of organic acids, and Goldfarb et al. (2011) found shifts in the active component of the microbial community for both glycine and sucrose. Again the shift was attributed to an increase in *r*-strategists (Goldfarb et al., 2011). Such changes caused by additions of LMW-C compounds are not isolated to the bacterial community but have also been noted for fungi (Hanson et al., 2008). There is therefore a growing body of research that shows changes in the composition of soil microbial communities are related to changes in the availability of LMW-C compounds. However, little evidence exists linking these compositional changes to functional changes, especially at input rates similar to those seen under field conditions.

A change in the function of soil microbial communities due to change in the inputs of LMW-C compounds is not an unlikely proposition. Specific groups of bacteria, such as those mentioned above, have been related to life history characteristics (i.e., *r*- vs. *K*-selection) that should also relate to their functional role (Fierer et al., 2007; Strickland et al., 2009b). Furthermore, change in the composition of the microbial community has been directly linked to change in the rates of ecosystem processes, such as litter decomposition (Strickland et al., 2009a,b). Additionally, a functional change within the community may be induced by LMW-C compounds without invoking a compositional change via shifts in community-level activity or physiology (van Hees et al., 2005). Thus, changes in the availability of specific LMW-C compounds, and the concomitant change this induces in the activity, physiology, or composition of the microbial community, may influence the function of that community.

A contrasting perspective on the impacts of LMW-C inputs on function is based on the fact that these compounds are inherently simple, not needing to be broken down via extracellular enzymes and in many instances are taken up directly by the microbial cell (van Hees et al., 2005). Barring competitive advantages or disadvantages incurred by interspecific differences in substrate affinity, this may instead mean that the role these compounds play in shaping the function of the microbial community may be rather limited and trumped by other factors. For instance, land-use legacies have been shown to have a marked influence on the mineralization and partitioning into various soil pools of glucose, a representative root exudate (Strickland et al., 2010a, 2012). Additionally, fertilizer regimes have been shown to influence the trophic transfer of C derived from a simple exudate compound (Lemanski and Scheu, 2014), raising the possibility that land use/management mediates how soil communities respond to LMW-C inputs. A definitive test as to the functional consequences of LMW-C compounds vs. other factors appears lacking. Yet, understanding whether change in inputs of LMW-C compounds is a dominant factor shaping the functional response of the soil microbial community is important because of the potential effect of environmental change on the amount and composition of root exudates (Drake et al., 2011; Phillips et al., 2011).

Here we test whether compositional differences in the inputs of LMW-C compounds induce a change in the function of microbial communities. We use realistic, low input rates (Hobbie and Hobbie, 2013), and add a factorial combination of three representative LMW-C compounds (glucose, glycine, and oxalic acid) applied across 90 days to soils originating from nine sites representing three land covers (mixed deciduous forest, white pine forest, or meadows). At the end of 90 days we assessed the function of these communities using an abbreviated catabolic response profile to determine which combinations of LMW-C compounds induced the greatest functional shift and whether land cover legacies mediate or even trump this response. From a sub-set of these samples, we also quantified whether 90 days of LMW-C compound addition induced a change in the microbial community composition using phospholipid fatty acid (PLFA) analysis. While the use of only three compounds fails to encompass the full complexity of actual root exudates, it does enable us to mechanistically examine the influence of three widespread constituents of root exudates, both individually and in combination (Luo et al., 2014). We ultimately address two questions: (1) What is the relative effect of contemporary additions of combinations of specific LMW-C compounds versus land-use legacies on soil microbial community composition and structure; and (2) Do specific land-use legacies, if present, mediate the effect of LMW-C compounds on soil microbial community composition and structure?

## Materials and Methods

### Site Description and Soil Collection

**FIGURE 1:**
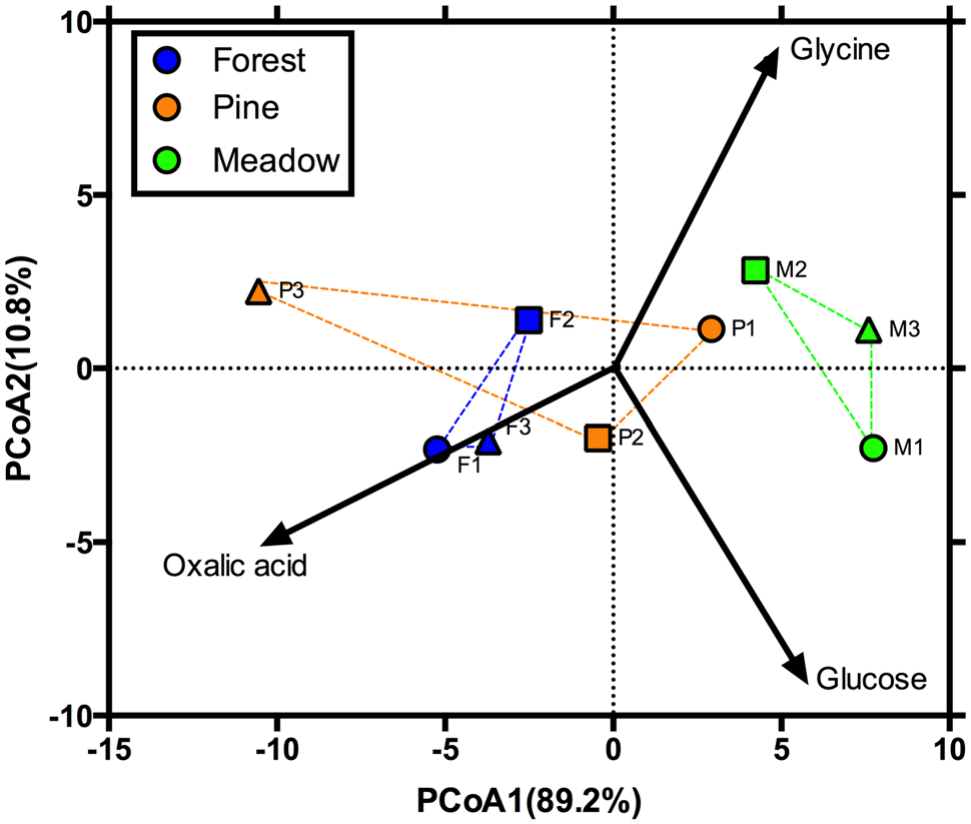
**Principal coordinates analysis (PCoA) showing the initial functional response of microbial communities.** Blue, orange, and green denote deciduous forests, pine stands, and meadows, respectively. Vectors illustrate increasing proportional mineralization of glucose, glycine, and oxalic acid.

In October 2009, soils were collected from nine sites representing three land cover types at Yale-Myers Forest, located in northeastern Connecticut, USA (41°56.98′N 72°7.08′W). The cover types included old-field meadows (hereafter referred to as meadows) dominated by *Poa* sp. and *Solidago* sp. (M1–M3), deciduous forests (hereafter referred to as forests) dominated by *Acer* and *Quercus* sp. (F1–F3), and white pine (*Pinus strobus*; hereafter referred to as pine) plantation stands (P1–P3; *n* = 3 in all cases giving a total of nine sites). We chose these sites and cover types because they are representative of the land cover of this region and these sites differ with regards to initial soil characteristics and microbial function, as determined via their catabolic response profiles (**Table 1**; **Figure 1**). Ten soil cores (0–10 cm depth) from each site were collected using a stratified random approach. Soils were then sieved (4 mm), homogenized, and stored at 5°C until use. Additionally, a subsample (∼10 g of soil) was stored at -80°C for PLFA analyses.

**Table 1:** Initial characteristics of the nine soils amended with simulated root exudates.

Site ID	Land-cover	Soil type	pH in water	Substrate induced respiration (SIR) [CO_2_-C (μg g dry wt soil^-1^ h^-1^)]	Mineralizable C [CO_2_-C (μg g dry wt soil^-1^)]
F1	Deciduous forest	Ridgebury, fine sandy loam	5.19	1.73	36.20
F2	Deciduous forest	Nipmuck-Brookfield complex, fine sandy loam	5.53	1.16	28.59
F3	Deciduous forest	Paxton and Montauk, fine sandy loam	4.90	1.63	49.43
P1	White pine	Paxton and Montauk, fine sandy loam	5.71	1.20	31.48
P2	White pine	Nipmuck-Brookfield complex, fine sandy loam	5.50	1.18	32.83
P3	White pine	Paxton and Montauk, fine sandy loam	5.00	2.19	53.65
M1	Meadow	Woodbridge, fine sandy loam	6.52	1.06	32.87
M2	Meadow	Paxton and Montauk, fine sandy loam	6.36	1.21	31.09
M3	Meadow	Woodbridge, fine sandy loam	6.56	1.28	29.86

The initial function (i.e., determined via catabolic response profile; see below), pH, active microbial biomass, and mineralizable C were assessed for each soil. We determined pH (1:1 soil: H_2_O by volume) using a benchtop pH meter. Active microbial biomass was determined via substrate induced respiration (SIR) following Strickland et al. (2010b). Briefly, 4 g of dry weight equivalent soil were combined with excess substrate (i.e., autolyzed yeast) creating a soil slurry that was pre-incubated for 1 h, followed by a 4 h incubation at 20°C. After 4 h, respiration was determined on an infrared gas analyzer (IRGA; Model LI-7000, Li-Cor Biosciences, Lincoln, NE, USA) using a static incubation technique. Mineralizable C was determined using a short-term 6-d incubation on soil from each site maintained at 65% water-holding capacity and 20°C with respiration across this time period determined using the technique described above for SIR. Total mineralizable C was estimated by integrating CO_2_ production across time.

### Simulated Root Exudate Experiment

Root exudates were simulated using three LMW-C compounds: glucose, oxalic acid, and glycine. Each of these compounds is representative of one of the three major classes of compounds found in root exudates [i.e., sugars, organic acids, and amino acids, respectively (de Graaff et al., 2010)]. These compounds, in addition to water only, were added in solution weekly for 90 days (to simulate one season worth of inputs) to 50 g of dry weight equivalent soil contained in plastic pots in the following combination: glucose only, glycine only, oxalic acid only, glucose and glycine, glucose and oxalic acid, glycine and oxalic acid, all three compounds, and water only (i.e., no addition of LMW-C). While the use of an artificial root system may have better simulated the spatial patterning and timing of exudation, the addition of low concentration, weekly doses likely minimized artifacts often associated with this type of application (e.g., unrepresentatively high substrate concentrations). Specifically, there were 72 experimental units [eight simulated exudate treatments (i.e., glucose, glycine, oxalic acid, all the combinations of these three compounds and water only) × nine sites representative of three land covers = 72]. Soils were amended with simulated root exudates at a rate of 45 μg of C g dry weight soil^-1^ year^-1^. This rate was derived from Phillips et al. (2008) assuming a bulk density of 1.32 g soil cm^-3^. Overall for each experimental unit, a total of 43.56 μg of C was added per week (0.87 μg of C g dry weight soil^-1^) and over the entire 90-days experiment ∼560 μg of C was added (∼11 μg of C g dry weight soil^-1^). During the entire course of this experiment soils were stored at 20°C and moisture was maintained at 65% water holding capacity. Prior to addition, simulated exudates were adjusted to pH 6 using NaOH or HCl. We expected that these additions of simulated root exudates would provide a strong test of the role of broadly representative root exudate compounds in structuring the function of soil microbial communities at relevant input rates to field soils (van Hees et al., 2005).

Within 1 week after soils were collected from the field (i.e., the initial sample), and also after 90 days of additions of simulated root exudates, we determined the functional response of the microbial community using a modified catabolic response profile similar to Degens and Harris (1997). We chose 90 days post simulated exudate additions because this would be equivalent to approximately one growing season’s worth of change in exudation patterns. By examining the function of the initial soils, we could determine what the effect of the differing treatments was after 90 days and the similarity/dissimilarity of these treatments to communities in the field. Functional assays consisted of amending 4-g dry weight equivalent soil with 8-ml solutions of glucose, glycine, or oxalic acid. Each compound (pH adjusted to six using NaOH or HCl) was added as 60 μg of C g dry weight soil^-1^. This addition amount was about an order of magnitude greater than the application rate per gram soil across the entirety of the 90-days incubations. As such, substrate limitation was removed and this specific rate was determined via a series of preliminary experiments to generate the maximum differences in induced respiration between soils. After a 1-h pre-incubation with shaking, the soil slurries (i.e., soil and solution combinations) were incubated for 4 h at 20°C. After incubation, respiration for each amendment was determined via IRGA of headspace CO_2_ concentrations.

**FIGURE 2:**
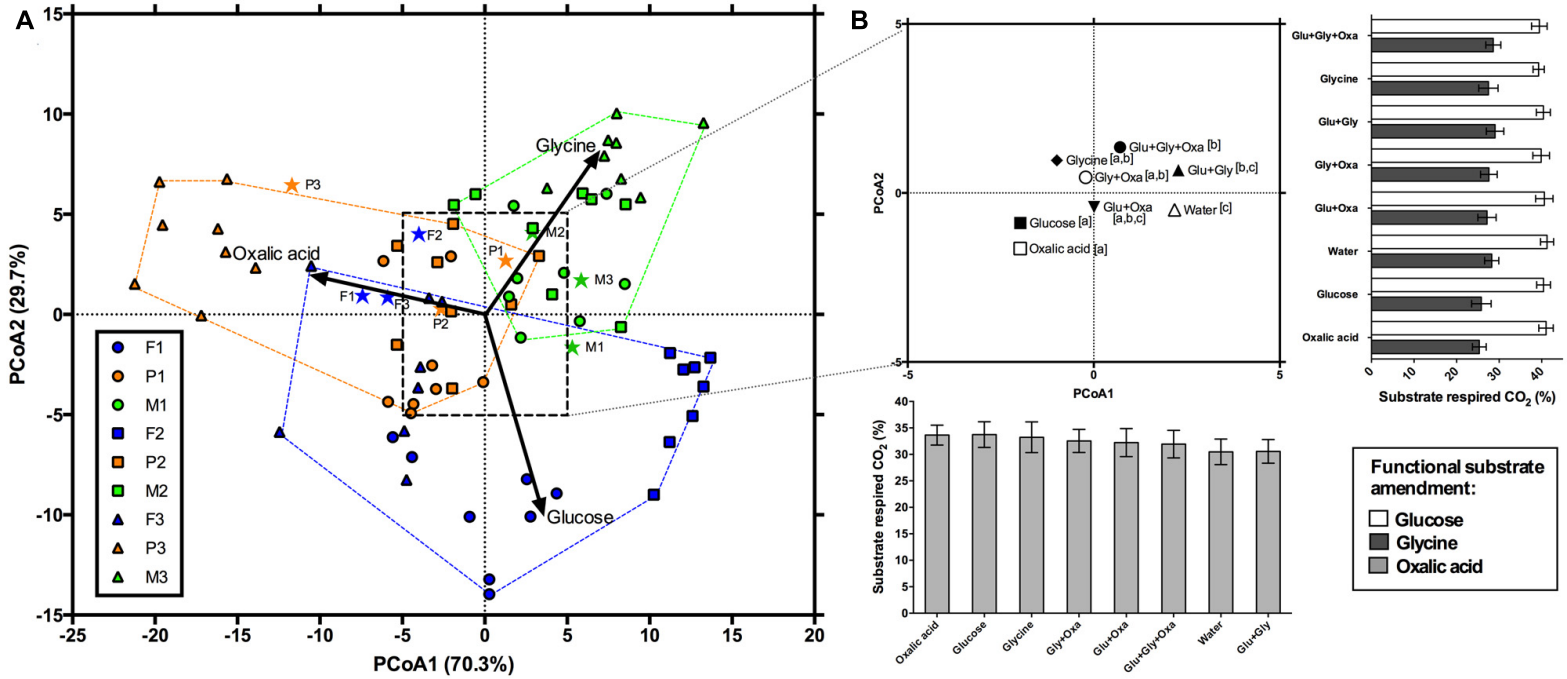
**Principal coordinates analysis showing the functional response of microbial communities after 90 days of exposure to simulated root exudates. (A)** The functional response of each land cover (blue, orange, and green are deciduous forests, pine stands, and meadows, respectively) and individual sites within each land cover, denoted by circles, squares, and triangles. Vectors illustrate increasing proportional mineralization of glucose, glycine, and oxalic acid. Stars represent the initial functional response of these communities. **(B)** An inset showing the centroids of the simulated exudate treatments. Letters denote pair-wise comparisons. The bar plot along the x-axis shows the change in proportional respiration (mean ± 1S.E.) of oxalic acid along PCoA1 and the bar plot along the y-axis shows the change in proportional respiration of glucose (open bars) and glycine (closed bars) along PCoA2.

To explore any potential linkages between the composition of the microbial community and its functional response to additions of simulated root exudates, we examined the effect of the exudate treatments on the PLFA profiles of the microbial communities for a subset of the sites (F2, M3, and P3, eight exudate treatments each, *n* = 24). We chose these sites because they represented all three land covers and also exhibited the greatest difference in function between sites (**Figure 2**). PLFA extractions and enumeration were performed as per Findlay and Dobbs (1993). Briefly, ∼5 g of soil was extracted in a phosphate-buffered dichloromethane solution to remove phospholipids, which were then separated using silicic acid chromatography, and derivatized in an alkaline solution to form fatty acid methyl esters (FAMEs). FAMEs were purified and quantified on a gas chromatograph equipped with a flame ionization detector (Shimadzu 2014 GC, Shimadzu Corp., Japan) and a Restek Rtx-1 column (Restek Corp., Bellefonte, PA, USA). FAMEs were identified and concentrations calculated based on a Supelco-37 component FAME mix standard (Sigma–Aldrich Co., St. Louis, MO, USA). Analyses were conducted on the relative abundance of FAMEs present at greater than 1%.

### Data Analysis

Results from the functional assays were standardized as a proportion of total substrate derived respiration. This standardization (i.e., proportional respiration) follows Degens and Harris (1997) and is calculated, for a given sample, by dividing the functional response of an individual compound by the sum of the functional responses for all three compounds after subtraction of respiration from controls that were amended with only water. We standardized by proportion of total substrate because we were interested in detecting functional shifts (i.e., catabolic response profiles) in the microbial communities, as opposed simply to increases or decreases in absolute respiration rates. We analyzed Euclidean distance matrixes constructed using the proportional respiration via a permutational MANOVA (perMANOVA). To examine the effect of the simulated root exudates on the functional response, we examined the interaction between the simulated exudate treatments and land cover while constraining the permutations within site nested in cover (this is the same as blocking the treatments by the site that the soils were collected from). This nesting allowed us to examine the effect of change in simulated exudates without the confounding influence of site level differences. Pairwise comparisons between exudate treatments were also analyzed via perMANOVA. We also tested for homogeneity of dispersions from the centroids for both the simulated root exudate and land cover treatments. Testing for homogeneity of dispersion allowed us to ascertain whether treatments were equally distant from their respective centroids and whether certain treatments exhibited greater variation in function. After perMANOVA analysis of PLFA profiles, we determined which PLFAs contributed the most to differences between land cover and treatment by determining the contribution of each PLFA to difference in Euclidean distance between groups. All perMANOVA analyses and tests of homogeneity were conducted using Primer (Clarke and Gorley, 2006).

To visualize the influence of the simulated exudate treatments and land cover on microbial community function and composition we used Principal Coordinates Analysis (PCoA). PCoA was conducted using the freeware statistical package (http://cran.r-project.org/)(R Development Core Team, 2012). Additionally, to determine the influence of initial site and community characteristics, we assessed whether pH, SIR, or mineralizable C were significantly related to microbial function after 90 days using regression analysis of these initial site characteristics and the first two axes of the PCoA. Because there were only nine sites, we used the site centroids for this analysis.

## Results

Permutational MANOVA indicated significant main effects of land cover, site, and exudate amendments, as well as a significant interaction between the exudate amendments and land cover (**Table 2**). For significant land cover effects (*F*_2,42_ = 3.4; *P* < 0.05), we found that the functional differences tended to be driven by greater proportional mineralization of glucose for the forest soils, glycine for the meadow soils, and oxalic acid for the pine soils (**Figure 2A**). This pattern of use did change after 90 days of incubation, but only slightly from that observed initially for these land covers (**Figures 1** and **2**). This slight change from initial land cover differences was primarily driven by increased proportional mineralization of glucose and glycine, and decreased proportional mineralization of oxalic acid for the forest soils; increased proportional mineralization of glycine and oxalic acid, and decreased proportional mineralization of glucose for the meadow soils (primarily due to site M3); and relatively little change over the 90 days period in the mineralization pattern of these compounds for the pine soils. We also noted significant differences in the homogeneity of dispersions from the centroids amongst the land cover treatments (*F*_2,69_ = 15.36; *P* < 0.001). This was due to the meadows exhibiting less dispersion (i.e., less variation in function across sites and treatment combinations) than either the forests or pine stands after 90 days of experimental treatments (**Figure 2A**).

**Table 2:** Permutational MANOVA (per MANOVA) examining change in microbial community function due to 90 days of simulated exudates (treatment) and land cover type.

Source of variation	df	SS	%SS	MS	*F*-value	*P*-value
Exudate treatment	7	239.1	3.1	34.2	3.1	**<0.01**
Cover type	2	3529.1	46.4	1764.5	3.4	**<0.05**
Site	6	3085.1	40.5	514.2	46.7	**<0.01**
Treatment × Cover	14	295.2	3.9	21.1	1.9	**<0.05**
Residuals	42	462.6	6.1	11.0		

*Note that while there were statistically significant effects of the exudate treatments, these effects explained very little variation as indicated by the percentage sums of squares (%SS). Title columns are degrees of freedom (df), sums of squares (SS), and means square (MS). Bold values denote significant P-values.*

For the simulated exudate treatments, significant differences in function were noted at the end of 90 days (*F*_7,42_ = 3.1; *P* < 0.01). Many of these effects appear due to pairwise differences between the treatment receiving no LMW-C additions (i.e., water only) and those treatments receiving some form of LMW-C addition (**Figure 2B**). Those treatments receiving only water for 90 days tended to exhibit the lowest average proportional mineralization of oxalic acid (30.48 ± 2.42%), and the greatest average mineralization of glucose (41.26 ± 1.53%). Those treatments receiving additions of just oxalic acid or glucose for 90 days tended to exhibit the greatest differences from those treatments that received some other combination of LMW-C or the water only treatment (**Figure 2B**). Compared to the other treatments, both the glucose and oxalic acid treatments tended to exhibit some of the greatest proportional respiration on oxalic acid (>33%) and the lowest proportional respiration on glycine (<25%). No significant differences in homogeneity of dispersions from the centroids of the exudate treatments were detected (*F*_7,64_ = 0.24; *P* = 0.98), suggesting that exudate treatments did not differentially affect variation in function.

There was a significant interaction between land cover and the simulated exudate treatments (*F*_14,42_ = 1.9; *P* < 0.05). Investigation of this interaction via a series of *post hoc* per MANOVA analyses, revealed that it was driven by significant effects of the simulated exudate treatments for both the forests (*F*_7,14_ = 2.64; *P* < 0.05) and meadows (*F*_7,14_ = 3.01; *P* < 0.01), but not the pine stands (*F*_7,14_ = 1.49; *P* = 0.18). That is, the forest and meadow communities did respond functionally to the addition of simulated exudates, but the pine communities did not. While difficult to disentangle pair-wise comparisons due to only three sites per land cover, for the forests differences in function seem to be due to the following pairwise differences: glycine vs. glucose, glycine vs. all three compounds, glycine vs. no addition, glucose + oxalic acid vs. all three compounds, and glucose + oxalic acid vs. no addition (*P* < 0.10 in all instances). For the meadows, the following pairwise differences were noted: oxalic acid vs. glucose + glycine, oxalic acid vs. all three compounds, glycine + oxalic acid vs. no addition (*P* < 0.10 in all instances).

**FIGURE 3:**
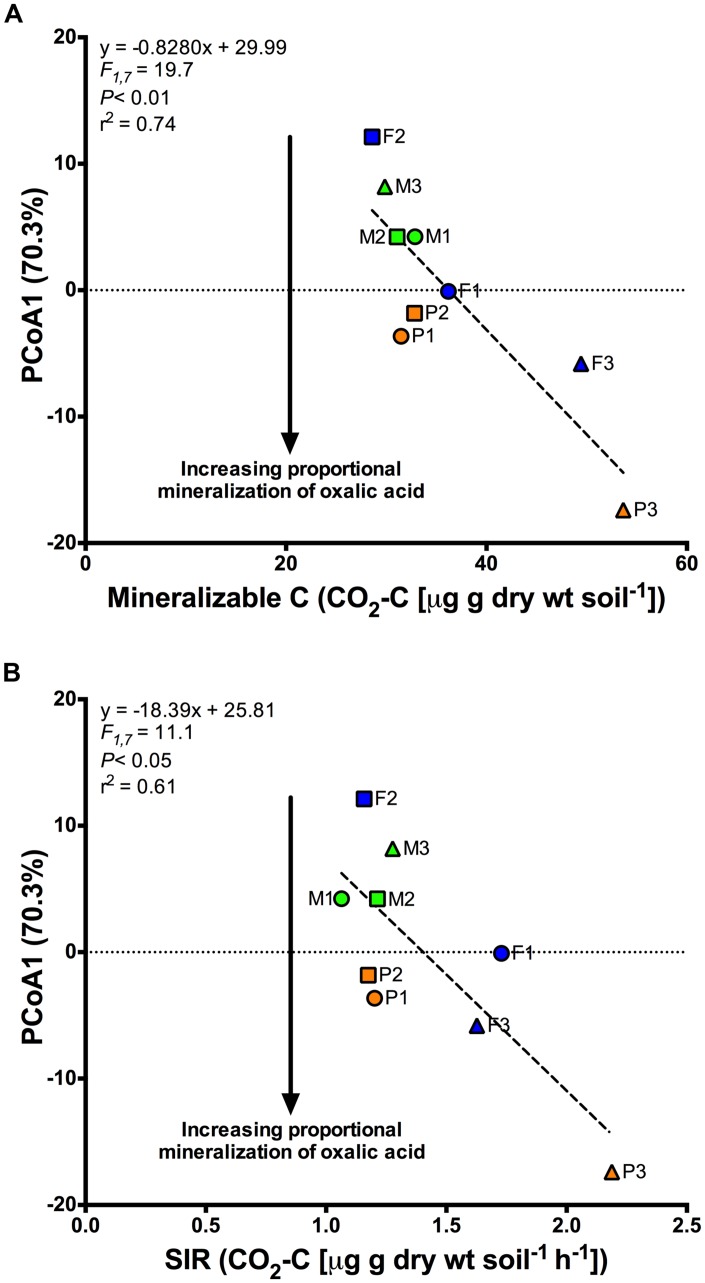
**Regression analyses showing the significant relationship between site level centroids along the first axis (PCoA1) of the principal coordinate analysis shown in **Figure 2A** and the initial values for (A) Mineralizable C, and (B) Substrate induced respiration (SIR), a metric of active microbial biomass.** PCoA1 was indicative of the proportional mineralization of oxalic acid after 90 days of exposure to simulated root exudates, with more negative values indicating greater mineralization and is indicated by the arrows shown in both figures.

Finally, with regards to function, even after 90 days of exudate amendments, site-level differences were still apparent (**Figure 2A**). To investigate whether initial site characteristics invoked this legacy effect, we assessed the relationship between site-level function using the site centroids and a suite of initial site characteristics via regression analysis of the first two axes of the PCoA (**Figure 3**). We found that SIR microbial biomass (*F*_1,7_ = 11.1; *P* < 0.05; *r*^2^ = 0.61) and mineralizable C (*F*_1,7_ = 19.7; *P* < 0.01; *r*^2^ = 0.74) were negatively related to the first principal coordinates axis. Proportional respiration of oxalic acid was most strongly related to this axis (increasing with decreasing axis values). This indicates that greater initial values of either SIR or mineralizable C were related to subsequently greater proportional mineralization of oxalic acid after 90 days of simulated exudate treatments (**Figure 3**).

**FIGURE 4:**
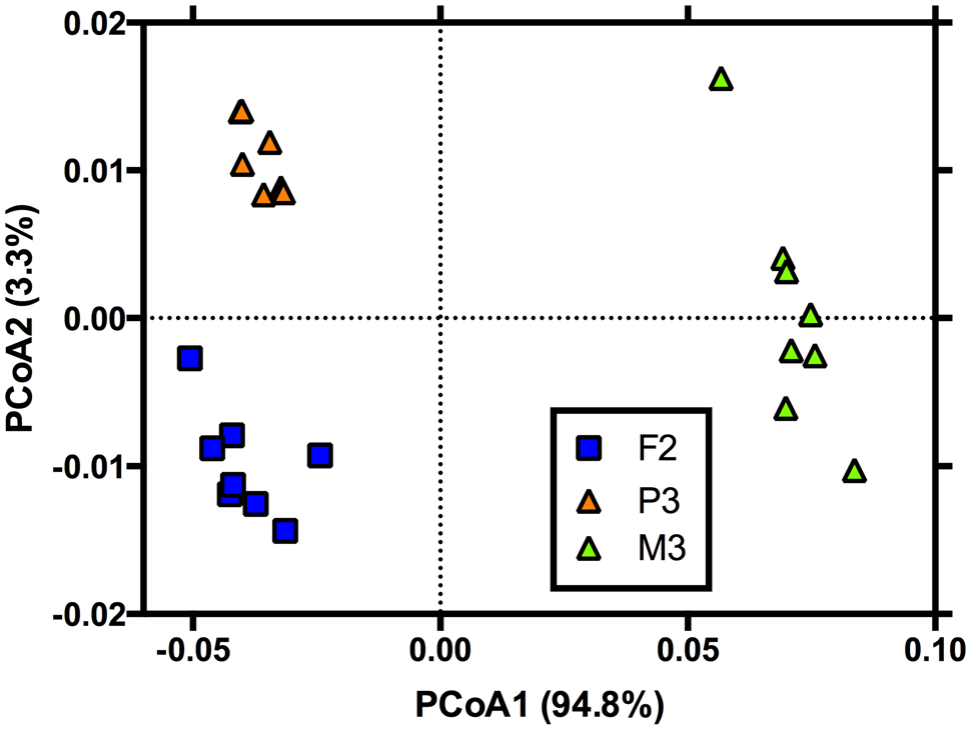
**Principal coordinates analysis showing the phospholipid fatty acid (PLFA) profiles of microbial communities after 90 days of exposure to simulated root exudates.** Land cover is denoted as in **Figure 1**. Note, the PLFA profiles were only assessed for one site in each land cover and were determined because these sites represented the most extreme differences in functional response after 90 days (**Figure 2**).

For microbial community composition assessed via PLFA analysis, we found that the simulated exudate treatments had no effect on community composition after 90 days (*F*_7,13_ = 1.19; *P* = 0.33) but that land cover was significantly related to composition (*F*_2,13_ = 280; *P* < 0.01). No significant differences in homogeneity of dispersions from the centroids for land cover were noted (*F*_2,20_ = 1.78; *P* = 0.31). For land cover, all three covers differed from one another (**Figure 4**). The land cover effects tended to be driven by a greater abundance of PLFA marker cy19 (Gram-negative bacteria) in the forest and pine sites vs. the meadow site. Further, the pine site tended to have a greater relative abundance of 10Me16 (Gram-positive bacteria/actinomycetes) and 16:1n7c (Gram-negative bacteria) and a lesser abundance of a15 (Gram-positive bacteria) versus the forest site (**Figure 4**).

## Discussion

The aim of this research was to determine the effects of LMW-C compounds, typically found in root exudates, on the function of soil microbial communities sourced from three common land cover types. To do this we amended soils for 90 days with combinations of three representative LMW-C compounds: glucose, glycine, and oxalic acid. While each of these compounds represents major classes of LMW-C compounds found in root exudates, these compounds have also been shown to markedly differ in microbial efficiency (Bradford et al., 2013; Frey et al., 2013). For instance, Frey et al. (2013) found that the microbial efficiency for glucose was ∼65% greater than that of oxalic acid and Bradford et al. (2013) found that the incorporation of glucose into microbial biomass was nearly three times that of glycine. After 90 days, we found that the experimental additions of LMW-C compounds were significantly – at least in a statistical sense - related to community function as assessed via a modified catabolic response profile (**Table 2**; **Figure 2**). We expected that additions of LMW-C could either have a marked impact on both community structure and function, have a marked impact on either structure or function, or have no impact on structure and function, when compared to land cover and management legacies. While these additions did shift function, but not structure, this shift was very slight overall.

We found that communities receiving amendments of only oxalic acid for 90 days tended to exhibit greater subsequent proportional respiration for oxalic acid when functional response profiles were assessed at the end (versus the start) of the experiment. This may suggest that these communities were conditioned to use oxalic acid, similar to the “home-field advantage” phenomenon observed for leaf litter (Strickland et al., 2009a). Yet it should be noted that the overall proportional increase in respiration was only ∼3% greater on average when compared to the simulated exudate treatment with the lowest proportional respiration for oxalic acid (i.e., glucose + glycine). Further, those soils amended with just glucose or glycine for 90 days had comparable proportional respiration of oxalic acid to those amended with just oxalic acid (**Figure 2B**). Hence, although the functional response profiles were affected by the composition of the LMW-C solutions they received, the main effects of this treatment on function appeared minor from the context of biological significance.

There was a significant interaction between treatments of LMW-C compounds and land cover. Functional differences between LMW-C treatments in the meadow and forest but not the pine sites drove this interaction, suggesting that the effects of LMW-C compounds on function may be mediated by land cover. This interaction could indicate that some communities may be more functionally plastic, taking advantage of change in the composition of LMW-C, while others are more functionally resistant. What shapes these functional attributes and/or what they might mean when considering change in the composition of LMW-C compounds entering systems is unknown, although our results suggest they might be minor.

While there is the possibility that change in LMW-C composition may alter the function of microbial communities in the longer-term, our findings suggest that in the short-term these changes may have minor ecological significance. Specifically, the additions of LMW-C compounds only accounted for ∼3% of the variance in function compared to the ∼46 and 41% of the variance explained by land cover type and site, respectively (**Table 2**). As such, factors associated with land cover and even site specific differences, such as recalcitrant litter inputs, nutrients, or pH (Strickland et al., 2009b, 2010a; Keiser et al., 2011), appear much more important for shaping microbial community function than LMW-C compounds despite the importance of the latter for fuelling heterotrophic soil respiration. In fact, we found that sites that initially exhibited greater SIR biomass or mineralizable C tended to also exhibit greater proportional mineralization of oxalic acid, including after 90 days of artificial root exudate amendments (**Figure 3**). This suggests that microbial activity and available soil C may be a stronger indicator of community function than are contemporary exudate C inputs. That is, microbial community function is not just a product of current conditions, if at all, but instead is a product of the legacy of cover and site conditions, likely constrained by the geology a soil experiences.

The treatment that showed the greatest difference from all other LMW-C treatments was the water-only control (**Figure 2B**). This may indicate that most LMW-C compounds can be used by a wide array of organisms and require little to no specialized mechanisms for uptake or degradation, in contrast to requirements for mineralization of more complex C compounds (van Hees et al., 2005; Eilers et al., 2010). Alternatively, the small change in function that we observed might be attributed to the low addition rates used in our study. We used an addition rate of 0.01 μg C g dry wt soil^-1^ day^-1^, whereas others have used rates ranging from 240 (Eilers et al., 2010) to 300 μg C g dry wt soil^-1^ day^-1^ (Shi et al., 2011). Such high addition rates of LMW-C compounds have recently been called into question (Hobbie and Hobbie, 2013); those used in our study were intended to more closely mimic natural conditions. Our results therefore suggest that environmental conditions that alter the composition of LMW-C compounds, such as various plant stressors like mineral nutrient availability and temperature (Badri and Vivanco, 2009), may have little direct impact on *in situ* microbial community function. Further research is needed to validate our conclusions, where both the quantity and composition of LMW-C compounds are manipulated for extended periods of time and the influence on a broad array of functions are assessed.

Not unlike function, we found that additions of LMW-C compounds had little effect on community composition as assessed by PLFA (**Figure 4**). Again, this may suggest the widespread ability of organisms to use LMW-C compounds (van Hees et al., 2005; Eilers et al., 2010). It also suggests the possibility that minor shifts in function due to variation in inputs of LMW-C are not linked to composition and that other mechanisms, such as soil pH (Fierer and Jackson, 2006), may be a more important compositional determinant. While we did not observe compositional shifts due to inputs of LMW-C, others have noted changes in the microbial community (Eilers et al., 2010; Shi et al., 2011). One explanation for this discrepancy is that many of these studies assessed change in composition via target gene sequencing approaches, such an approach may have greater taxonomic resolution compared to PLFA. Another explanation, similar to function, is the much greater amount of LMW-C added in those previous experiments. If the expectation is that LMW-C compounds are entering systems at much lower rates than previously simulated (Hobbie and Hobbie, 2013), then our observations may be more in line with those effects likely to occur in nature.

## Conclusion

While we did note some changes in community function associated with different combinations of simulated exudates, such changes may have relatively little ecological significance. However, further research should examine the potential role additional compounds found in root exudates play in determining function. The legacy of land cover and site specific differences appear to have a much greater influence on community composition and function, despite the highly controlled nature of our experimental simulations. Notably, change in the composition of the inputs played less of a role in shaping function as compared to a lack of exudates. That is, community function was maintained as long as LMW-C compounds were added. Our results, together with the growing realization that LMW-C enters soils at low rates (at the microscale), suggests that compositional changes in exudate patterns may have little impact on community structure and function despite the dominant role these compounds play in determining absolute respiration rates.

## Conflict of Interest Statement

The authors declare that the research was conducted in the absence of any commercial or financial relationships that could be construed as a potential conflict of interest.
